# Emerging Metabolic Targets in the Therapy of Hematological Malignancies

**DOI:** 10.1155/2013/946206

**Published:** 2013-08-18

**Authors:** Zaira Leni, Geetha Parakkal, Alexandre Arcaro

**Affiliations:** University of Bern, Department of Clinical Research, Division of Pediatric Hematology/Oncology, Murtenstrasse 31, 3010 Bern, Switzerland

## Abstract

During the last decade, the development of anticancer therapies has focused on targeting neoplastic-related metabolism. Cancer cells display a variety of changes in their metabolism, which enable them to satisfy the high bioenergetic and biosynthetic demands for rapid cell division. One of the crucial alterations is referred to as the “Warburg effect”, which involves a metabolic shift from oxidative phosphorylation towards the less efficient glycolysis, independent of the presence of oxygen. Although there are many examples of solid tumors having altered metabolism with high rates of glucose uptake and glycolysis, it was only recently reported that this phenomenon occurs in hematological malignancies. This review presents evidence that targeting the glycolytic pathway at different levels in hematological malignancies can inhibit cancer cell proliferation by restoring normal metabolic conditions. However, to achieve cancer regression, high concentrations of glycolytic inhibitors are used due to limited solubility and biodistribution, which may result in toxicity. Besides using these inhibitors as monotherapies, combinatorial approaches using standard chemotherapeutic agents could display enhanced efficacy at eradicating malignant cells. The identification of the metabolic enzymes critical for hematological cancer cell proliferation and survival appears to be an interesting new approach for the targeted therapy of hematological malignancies.

## 1. Introduction

This paper will review a variety of important aspects of metabolic processes relevant to cancer development and maintenance, with a focus on haematological malignancies (HMs). In addition, we will highlight small molecule compounds that inhibit glycolysis and other interconnected processes and their potential applications in HMs treatment. 

Over the past decades, many research groups have commonly reported that one of the main features of tumor cells is to bear a variety of mutations that combine to redirect the network of intracellular signalling pathways. Moreover, modern high-throughput DNA sequence analysis has suggested that these mutations are more numerous and heterogeneous than previously thought [1, 2]. In some cases, the mutations differ among histopathologically identical tumors and are altered during the process of tumor progression [3]. As a consequence, tumor development and progression are increasingly considered to be extremely complex processes. Thus, in most cases, it will be difficult or impossible to specifically eradicate cancer cells by targeting a single oncogene. Hence, further insights into the biological differences between cancer cells and normal cells are necessary to design and develop novel selective and effective anticancer therapies. However, it is becoming extensively clear that many oncogene-activated signalling pathways converge towards an adaptation of tumor cell metabolism to provide energy and essential biomolecules required for the rapid cell division [4–6]. Within the last years, a large variety of different solid tumors have been associated with increased metabolism (i.e., prostate cancer [7], breast cancer [8], glioblastoma [9], ovarian carcinoma [10], pancreatic cancer [11], and many others). It is now accepted that the metabolism of cancer cells has extremely unique characteristics compared to the one of healthy nonproliferating cells [4]. Indeed, cancer cells display a metabolic adaptation called aerobic glycolysis or “Warburg effect”, which consists of a metabolic shift to increase the glycolytic pathway as a main source of ATP, instead of oxidative phosphorylation (OXPHOS), independent of the presence of oxygen [12, 13]. Several studies have demonstrated that this shift massively contributes to satisfying the high bioenergetic and biosynthetic demands for rapid cell division in cancers [12–14]. In addition, high rates of glycolysis in tumor cells have been related to resistance to chemo- and radiotherapy treatment [15]. These observations suggest that blocking glycolysis may negatively affect tumor progression and may enhance the efficacy of chemo- and radiotherapy. Indeed, a variety of studies performed *in vivo* (i.e., human osteosarcoma, lung carcinoma [15], and ovarian cancer [16]) and *in vitro* (i.e., glioma, squamous carcinomas, and colon carcinoma cells [17]) have revealed that targeting the glycolytic pathway with specific compounds increases the cellular sensitivity toward commonly used anticancer drugs. 

## 2. Glycolysis

**Figure 1:**
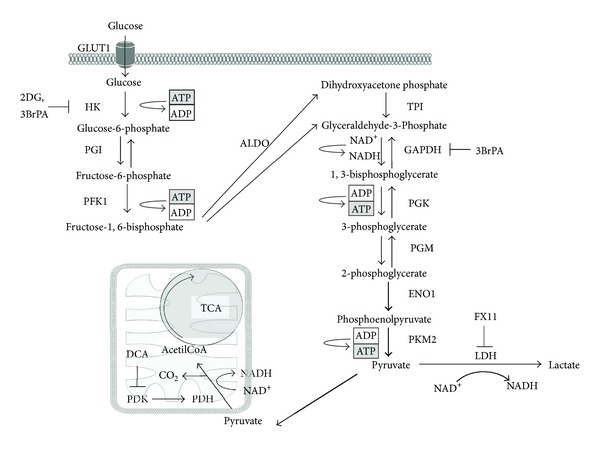
Glycolysis and molecular targets of glycolytic inhibitors. The solid arrows indicate glycolytic reactions. 2-Deoxyglucose (2-DG) inhibits hexokinase (HK), inducing early blockage of glycolytic pathway. 3-Bromopyruvate (3BrPA) inhibits HK and glyceraldehyde-3-phosphate dehydrogenase (GAPDH), both blocking glycolytic flux. [3-dihydroxy-6-methyl-7-(phenylmethyl)-4-propylnaphthalene-1-carboxilic acid] (FX11) inhibits lactate dehydrogenase (LDH), further preventing the lactate production. Dichloroacetate (DCA) inhibits pyruvate dehydrogenase kinase (PDK), limiting the acetyl-CoA production by pyruvate dehydrogenase (PDH). GLUT1, glucose transport 1; HK, hexokinase; PGI, phosphoglucose isomerase; PFK, phosphofructokinase; TPI, triosephosphate isomerase; GAPDH, glyceraldehyde-3-phosphate dehydrogenase; PGK, phosphoglycerate kinase; PGM, phosphoglycerate mutase; ENO1, enolase; PK, pyruvate kinase; LDH, lactate dehydrogenase; PDH: pyruvate dehydrogenase; PDK, pyruvate dehydrogenase kinase.

Glycolysis is a 10-step pathway that converts a glucose molecule into 2 pyruvates with a net production of 2 ATP molecules and in parallel provides intermediates for anabolic pathways (Figure 1). Each step of the glycolytic reaction is catalyzed by a specific enzyme or enzyme complex. Some of these enzymes comprise isoform variants that are expressed in a context- and tissue-dependent manner [18], leading to enhanced complexity in the glycolytic pathway. Different glycolytic proteins have been reported to be deregulated in various cancers, thus contributing to aerobic glycolysis (Table 1).

## 3. Glucose Transporters

The entry of glucose into cells is achieved via facilitated diffusion through the glucose transporters (GLUT) family [18]. This family consists of 14 proteins grouped in 3 subclasses, which differ from one another in their affinity for glucose [19]. GLUT1 promotes elevated rates of glucose transport into cells, and the deregulation of its expression has been reported in many tumours, but not in normal tissues [20].

## 4. Hexokinase 

Hexokinase (HK) comprises four isoforms (I to IV), which differ in their kinetics proprties, as well as in their tissue-specific expression and subcellular localization [21, 22]. The hexokinase isoform II (HKII) is known to play a crucial role in initiating and maintaining the high glucose catabolic rates of rapidly growing tumors [23, 24]. Certain tumor cells display an increased gene copy number in *HKII* [25]. The *HKII* gene promoter is the target of multiple signals activated by glucose, hypoxic conditions, and insulin, all of which enhance the rate of its transcription [26, 27]. Due to the binding of HKII to the mitochondrial outer membrane where the voltage-dependent anion channel (VDAC) is located [22], this enzyme uses the ATP produced by oxidative phosphorylation as a substrate to produce G-6-P. This interaction of HKII with the mitochondrial membrane appears to be tighter in tumor cells than in normal cells [28]. The HKII-VDAC interaction is thought to be the link between altered cellular metabolism and inhibition of apoptosis because it confers resistance against mitochondrial membrane permeabilization, which is a critical step in apoptosis [29]. 

## 5. Glucose-6-Phosphate Isomerase

The third glycolytic enzyme in the pathway is glucose-6-phosphate isomerase (PGI). This enzyme is used as a prognostic marker, as the expression of PGI is associated with cancer progression and an aggressive malignant behavior [30, 31]. However, the involvement of PGI in cancer metabolism has not been completely elucidated yet. 

## 6. Phosphofructokinase

The following enzyme in the pathway is phosphofructokinase (PFK), which is an important control point in the glycolytic pathway and is generally thought to maintain the glycolytic flux [32, 33]. PFK activity is markedly increased in cancer cell lines and primary tumor tissues *in situ *[34–36]. Elevated PFK-1 activity is also characteristic of cancer cells and is induced in response to oncogenes or following hypoxia-inducible factor 1*α* (HIF1*α*) activation [21, 37]. It has been recently reported that the inhibition of PFK by posttranslational modification, such as glycosylation, confers a selective growth advantage to cancer cells [38].

## 7. Glyceraldehyde-3-Phosphate Dehydrogenase

Another enzyme associated with increased glycolytic activity is glyceraldehyde-3-phosphate dehydrogenase (GAPDH) [39, 40]. GAPDH affects multiple cellular processes including endocytosis, exocytosis, membrane fusion, vesicular secretory, neuronal apoptosis, DNA replication and repair, nuclear tRNA transport, and cytoskeletal organization [21, 41], due to its known nonenzymatic activities, such as binding to NAD^+^or NADH and also to DNA and RNA [35, 42–44].

## 8. Pyruvate Kinase

The final reaction of glycolysis is catalyzed by pyruvate kinase (PK), which has been reported to play a crucial role in reprogramming glycolytic metabolism. There are four mammalian PK isoenzymes (M1, M2, liver isoform (L), and a red blood cell isoform (R)). PKM2 is the embryonic and cancer-associated isoform and exerts its function by forming a tetramer or a less active dimer [45]. The less active dimeric form of PKM2 is mainly found in cancer. It is most commonly expressed in colon cancer [46], renal carcinoma [47], breast cancer [48], lung cancer, and gastrointestinal tumors [49]. PKM2 is known to directly contribute to the Warburg effect [46], as it contributes to the accumulation of glycolytic intermediates for the following anabolic processes: nucleic acid, amino acid, and phospholipid synthesis [50]. PKM2 imparts a growth advantage to tumor cells, particularly under hypoxic conditions. Replacement of PKM2 by the normal adult isoform PKM1 in tumor cells decreased their glycolytic rate and diminished their ability to grow as xenografts [46]. 

## 9. Lactate Dehydrogenase

Lactate dehydrogenase (LDH) is a tetrameric enzyme that exists in five isoforms, mostly located in the cytosol [51]. The five isoforms are made up of various possible combinations of the two types of subunits: LDH-A and LDH-B [52]. It has long been appreciated that many human cancers have higher levels of LDH expression than normal tissue [53, 54], and, therefore, LDH has already been acknowledged as one of the most promising cancer targets. In fact, the inhibition of LDH-A had an antiproliferative effect on breast tumor [55, 56]. In addition, studies in primary lymphoma of the breast suggest that aggressive behavior and elevated LDH levels are prognostic factors for poor prognosis and survival [57].

## 10. Tricarboxylic Acid Cycle 

Mitochondrial pyruvate dehydrogenase (PDH) converts pyruvate into acetyl-CoA, which is further directed into the tricarboxylic acid cycle (TCA cycle) and oxidative phosphorylation. PDH is negatively regulated by pyruvate dehydrogenase kinase (PDK) via phosphorylation [58]. This phosphorylation of PDH decreases the entry of pyruvate into the mitochondrial oxidative metabolism [59]. PDK is known to be a key regulator of the Warburg effect [60] and considered as a novel therapeutic target in oncology [61].

A broad range of cancer types appear to have an increased glycolytic pathway and to take advantage of using intermediates for anabolic reactions [62, 63]. Transcription factors such as HIF1*α* [64, 65], c-Myc [66–68], and p53 [69] contribute directly or indirectly to this metabolic adaptation of tumors. These interactions seem to be fundamental to direct the aberrant metabolic behavior of tumor cells by promoting the Warburg effect [67–70]. Nevertheless, the gene networks involved in cancer metabolism have not been completely elucidated yet. Thus, it would be beneficial to reverse the Warburg effect in order to normalize tumor metabolism, which may be a potential therapeutic strategy for the treatment of cancer.

Since one feature of HMs is their potential for rapid proliferation and high metabolic demand, a better understanding of the regulation of the multiple metabolic pathways in HMs may reveal new therapeutic opportunities, particularly by restoring the altered cancer energy metabolism. 

## 11. Preclinical Efficacy of Glycolytic Inhibitors

Glycolysis inhibitors have been usually developed to target enzymes that are deregulated in cancer cells compared to their normal counterparts. Many glycolytic inhibitors have been developed so far, and their efficacy has been demonstrated by both *in vitro* and *in vivo* studies [71]. Moreover, some of these inhibitors have already undergone clinical testing (Table 2). Although the exact molecular mechanisms underlying the reliance of tumors on glycolysis remain not completely understood [71], glycolysis inhibition opens feasible therapeutic windows for cancer treatment. Indeed, several small molecules have been identified and have been shown to exhibit promising anticancer activities both *in vitro* and *in vivo*, as single agents or in combination with other therapeutic modalities.

## 12. Hexokinase Inhibitors: 2-Deoxyglucose (2-DG)

**Figure 2:**
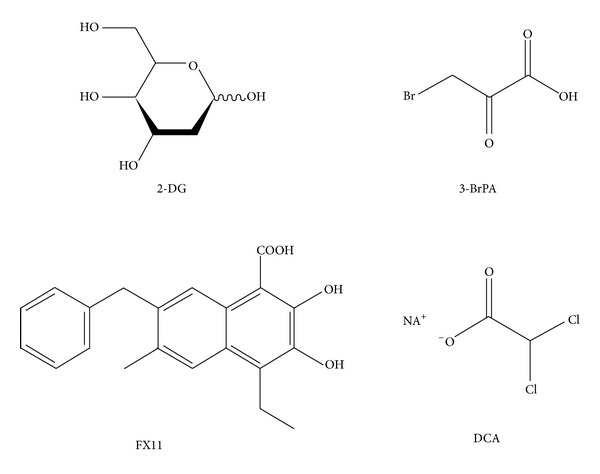
Chemical structures of glycolytic inhibitors. 2-Deoxyglucose (2-DG) and 3-bromopyruvate (3-BrPA) have been both applied in preclinical studies in HMs [110, 113, 121], whereas (FX11 [3-dihydroxy-6-methyl-7-(phenylmethyl)-4-propylnaphthalene-1-carboxilic acid]) (FX11) and dichloroacetate (DCA) have been applied in different solid tumors [92, 96].

2-Deoxyglucose (2-DG) (Figure 2) is an early glycolytic inhibitor, which has been proven to be effective at depleting cellular ATP [72, 73]. 2-DG is a glucose analogue, which is phosphorylated by the enzyme HK to 2-deoxyglucose-6-phosphate (2-DG-6-P). 2-DG-6-P cannot be further metabolized and accumulates in the cytoplasm, leading to a proximal blockade of glycolysis [73]. Several reports have shown that the cytotoxic effects of 2-DG are heterogeneous [74, 75]. The cytotoxic effects of 2-DG were found to be higher in cancer cells under hypoxic conditions or in cells with mitochondrial defects [76–79]. Recent studies have reported the therapeutic efficacy of 2-DG combined with chemotherapeutic drugs or ionizing radiation, and indeed, in both cases, the inhibitor enhanced the damage to cancer cells, while reducing the damage to normal cells [78, 79] (i.e., 2-DG in combination with cisplatin and doxorubicin had a significant cytotoxic effect in rapidly dividing cells, whereas no effect was seen in slowly growing cells [80].) 2-DG can also enhance DNA damage caused by irradiation in cancer cells [76]. 

## 13. Multiple Glycolytic Inhibitors: 3-Bromopyruvate (3-BrPA)

The metabolic blocker 3-bromopyruvate (3-BrPA) (Figure 2) is a halogenated analogue of pyruvic acid with strong alkylating properties and has received significant attention due to its remarkable antitumor activity [71, 81]. *In vitro* testing demonstrated that 3-BrPA inhibits glycolysis and blocks ATP production, thus causing apoptosis in a dose-dependent manner [71]. Like 2-DG, 3-BrPA exhibited a potent cytotoxic activity against cancer cells with mitochondrial respiratory defects and against cells in a hypoxic environment [82]. 3-BrPA is believed to inhibit HKII through a covalent modification at one or more cysteine residues leading to an attenuation of the glycolytic rate [83]. Further studies using radiolabelled 3-BrPA have identified the glycolytic enzyme GAPDH as the primary intracellular target of this agent [84]. The binding of 3-BrPA to GAPDH caused an inhibition of its enzyme activity and, consequently, of the glycolytic production of ATP, which led to cell death by apoptosis [85]. The antineoplastic effects of 3-BrPA resulted from a reduction in some intermediates of glycolysis, thus impairing the replenishment of anabolic reactions branching from the glycolytic pathway [86]. Indeed, the 3-BrPA-mediated inhibition of HKII could result in a reasonable reduction in ribose-5-phosphate synthesis [71]. Similarly, GAPDH inhibition could decrease the levels of its downstream metabolite, 3-phosphoglycerate, thus reducing the production of lipids and amino acids deriving from it [87]. 3-BrPA also displayed an effect on the extra-glycolytic enzyme succinate dehydrogenase (SDH) [88], which contributed to a block in ATP production and to an impairment of mitochondrial respiratory function. 3-BrPA is effective at a concentration of 100 nM, meaning that it is more potent than 2-DG, which is effective in the mM concentration range [88].

Although, many preclinical studies have confirmed its anticancer properties, the molecular targets and the mechanisms underlying 3-BrPA-induced cytotoxicity have not been completely defined thus, its application in human has not been tested by clinical trials yet. 

## 14. Inhibition of PKM2

Inhibition of PKM2 has so far been achieved by using RNA interference (RNAi), which induced apoptosis and tumor regression, partially by enabling a reversion of the metabolic shift [89].

## 15. LDHA Inhibitors: (FX11 [3-Dihydroxy-6-methyl-7-(phenylmethyl)-4-propylnaphthalene-1-carboxilic Acid])

FX11 [3-dihydroxy-6-methyl-7-(phenylmethyl)-4-propylnaphthalene-1-carboxilic acid] (FX11) (Figure 2) is a small-molecule inhibitor selective for LDHA [90]. FX11 was identified through screening a library of compounds derived from the natural product gossypol, a known antimalarial LDH inhibitor [91]. FX11 was shown to induce oxidative stress and cells death *in vitro*, as well as an inhibition of the progression of human lymphoma and pancreatic xenografts* in vivo* [92]. Additional studies have described a reduction in ATP levels and the induction of significant oxidative stress and cell death upon treatment with FX11 [92]. Several studies have documented that inhibition of LDHA can reduce cellular transformation and markedly delay tumor formation, indicating that LDHA is required for tumor progression [93, 94]. It has also been shown that FX11, when combined with FK866, another metabolic inhibitor that inhibits NAD^+^ synthesis through a direct inhibition of nicotinamide phosphoribosyltransferase, can induce lymphoma regression [95]. Nevertheless, the evaluation of FX11 in clinical studies has not been reported on yet. 

## 16. PDK1 Inhibitors: Dichloroacetate 

Dichloroacetate (DCA) is a mimetic form of pyruvate, and among several glycolytic inhibitors it is known to significantly decrease lactate production in myeloma cancer cell lines [96]. This is due to the ability of DCA to inhibit PDK1 [97–99], resulting in impaired phosphorylation of PDH. DCA treatment restored the activity of PDH which can supply acetyl-CoA to the TCA cycle and oxidative metabolism [98]. The antitumor activity of DCA was documented against a variety of cancer cells type derived from the lung [97], breast [100], prostate [101], endometrial [102], and colorectal cancers [103].

## 17. Hematological Malignancies

HMs comprise a collection of heterogenous diseases, all originating from cells from the bone marrow and the lymphatic system. The overall prevalence of haematological cancers is increasing representing the fifth most common cancer group [104]. HMs include lymphomas, leukemia, myeloproliferative neoplasms, plasma cell dyscrasias, histiocytic tumor, and dendritic cell neoplasms. The incidence of HMs increases with age (mean age at diagnosis: 63 years). There is no gold standard for hematopoietic disease classification thus; multiple methods were applied over the years. Among them, the most frequently used systems include Revised European-American classification in 1994 (REAL), French-American-British system (FAB), and World Health Organization (WHO) classification in 2001 (updated in 2008) [105]. The inability of the earliest classification, based on cellular morphology and histological architecture, to distinguish HMs subtypes led to the incorporation of two additional criteria into the REAL classification by the WHO in 2001: the immunophenotype and the genetic background.

Nowadays, the WHO classification updated in 2008 is widely used and accepted [106]. Indeed, this system classifies malignancies of the hematopoietic and lymphoid tissues based on morphological, immunophenotypic, genetic, and clinical features with the aim of increasing diagnostic accuracy. In addition, this classification has created borderline categories for cases that do not fit into a particular subgroup. When possible, the different cancer types are grouped by lineage into myeloid neoplasms, lymphoid neoplasms, and histocytic/dendritic neoplasms [105]. There are also neoplasms that show evidence of both myeloid and lymphoid differentiations, probably due to the fact that they are derived from multipotent progenitors cells, and these are then classified as neoplasms of myeloid and lymphoid lineages. 

## 18. Myeloid Neoplasms 

Myeloid neoplasms are usually derived from bone marrow committed progenitors restricted to give rise to erythrocytes, granulocytes (neutrophils, basophils, and eosinophils), monocytes, or megakaryocytes. Myeloid neoplasms are generally adult diseases presenting symptoms at a median age of 64 years. They are be subgrouped into three broad clinical classes: acute myeloid leukemias (AML), myeloproliferative neoplasms (MPN), and myelodysplastic syndromes (MDS). The first one, AML, has a very aggressive outcome and requires immediate therapy, whereas the clinical behaviour of MPN and MDS can be quite indolent. AML is characterised by more than 20% of myeloid blasts in the bone marrow or in the peripheral blood and is subdivided into five groups: (a) AML with recurrent genetic abnormalities, (b) AML with myelodysplasia-related changes, (c) therapy-related myeloid neoplasms, (d) AML not otherwise specified, and (e) myeloid sarcoma and myeloid proliferation related to Down syndrome. 

The second myeloid neoplasms, MPN, is a group of disorders associated with the proliferation of one specific myeloid lineage (i.e., granulocytes, erythroid, megakaryocytic, or mast cells). This disease is often associated with mutations causing abnormal increases in tyrosine kinase activity and growth factor-independent proliferation of bone marrow progenitors. The MPN can be divided into (a) chronic myeloid leukemia (CML), (b) chronic neutrophilic leukemia (CNL), (c) polycythemia vera (PCV), (d) essential thrombocythemia (ET), (e) primary myelofibrosis (PMF), (f) chronic eosinophilic leukemia (CEL), (g) mastocytosis, and (h) unclassificable myeoloproliferative neoplasms (referred to as atypical MPNs). The third subtype of myeloid neoplasms, MDS, refers to disorders exhibiting dysplasia and with a variable risk of transformation to acute leukemia. Furthermore, the hematopoiesis is ineffective and results in cytopenias. Like AML and MPN, MDS is composed of several subtypes: (a) refractory cytopenia with unilineage dysplasia, (b) refractory anemia with ring sideroblasts, (c) refractory cytopenia with multlineage dysplasia, (d) refractory anemia with excess blasts, (e) unclassifiable MDS, and (f) childhood MDS. 

## 19. Lymphoid Neoplasms

Lymphoid neoplasms are those derived from cells that develop into T lymphocytes (cytotoxic T lymphocytes, helper T lymphocytes, or regulatory T lymphocytes) or B lymphocytes. Lymphoid neoplasms are divided into 2 groups: neoplasms derived from lymphoid precursors (i.e., acute lymphoblastic leukemia/lymphoma, ALL) and neoplasms of mature lymphocytes and plasma cells. The first group is composed two main categories: precursor B lymphoblastic leukemia/lymphomas and precursor T lymphoblastic leukemia/lymphoma. Concerning the neoplasms of mature lymphocytes, the WHO grouped the diseases based on B or T cell origin. The classification of mature B cell neoplasms is based on a comparison of aberrant and normal B cell development and is divided into (a) chronic lymphocytic leukemia/small lymphocytic lymphoma (CLL/SLL), (b) lymphoplasmacytic lymphoma (LPL), (c) mantle cell lymphoma (MCL), (d) B cell prolymphocitic leukemia (B-PLL), (e) follicular lymphoma (FL), (f) diffuse large B cell lymphoma (DLBCL), (g) Burkitt lymphoma/leukemia (BL), (h) marginal zone B cell lymphoma (MZL), (i) hairy cell leukemia (HCL), and (l) plasma cell myeloma/plasmacytoma. Hodgkin's disease or Hodgkin lymphoma (HL) is separated from the other B cell lymphomas based on its unique cellular composition, containing a minority of neoplastic cells in an inflammatory background. HL can be divided into two major subgroups: nodular lymphocyte predominant HL and classical HL. 

The mature T cell and natural killer (NK) cell neoplasms are lymphoid neoplasms of mature T cell or NK cells including (a) peripheral T cell lymphoma (PTCL), (b) anaplastic large cell lymphoma (ALCL), (c) primary cutaneous peripheral T cell lymphomas, adult T cell leukemia-lymphoma (ATL), (d) T large granular lymphocyte leukemia (LGL), (e) T cell prolymphocytic leukemia (T-PLL), and (f) NK cell large granular lymphocyte leukemia (LGL).

The last group of HMs, according to the 2008 WHO classification, includes histiocytic/dendritic neoplasms derived from accessory antigen-presenting precursor cells (APC or dentritic cells) or connective tissue macrophages (histiocytes). Histiocytic/dendritic neoplasms are divided into 3 groups: (a) histiocytic sarcoma, (b) tumors derived from Langerhans cells, and (c) follicular dendritic sarcoma.

## 20. Risk Factors and Standard Protocol for HMs

There are many risk factors playing a role in HMs development. Age is the most significant risk factor, since these cancers most often occur in patients aged 60 or older. Furthermore, benzene exposure, ionizing radiation, or certain types of viral infections (i.e., HTLV1 or HIV) have been linked to the development of leukemia. The type of cancer, stage, age, sex, race, and the presence of chromosomal abnormalities are important factors in HMs treatment. Based on these factors, treatment may include chemotherapy, radiotherapy, targeted therapies, and hematopoietic stem cell transplantation. The frequent chromosomal abnormalities in HMs include gene deletions, amplifications, and translocations. The major and most important chromosomal abnormality in CML is the presence of the Philadelphia chromosome (Ph^+^) arising from a translocation t(9;22) resulting in the BCR/ABL (Breakpoint Cluster Region/ABelson murine Leukemia viral oncogene homolog) gene fusion. More than 85% of patients diagnosed with CML have the Ph^+^ chromosome. The presence of the Ph^+^ chromosome, in the absence of additional chromosomal changes, is associated with a good prognosis in CML. A large number of structural and numerical chromosomal changes have been described in ALL [107]. However, some of these changes occur more frequently than others. The human mixed lineage leukemia (MLL) gene on chromosome 4, the t(12;21) translocation resulting in the TEL/AML (Translocation Ets-Leukemia/Acute-Myeloid-Leukemia) gene fusion, hyperdiploidy, together with the t(9;22) translocation, which produces the BCR/ABL fusion, are the most common karyotypic abnormalities in ALL. The TEL/AML fusion gene appears to be associated with a good prognosis in ALL, while the presence of BCR/ABL has an unfavorable prognosis [108]. 

## 21. Targeting the Glycolytic Pathway in HMs

Although there are many examples of rapidly proliferating solid tumor cells that have a fundamental change in their metabolism and exhibit an increased dependency on the glycolytic pathway for ATP generation, it has been only recently reported that this phenomenon also occurs in HMs.The metabolic difference between normal cells and cancer cells provides a basis for the design of therapeutic approach to selectively kill cancer cells. So far, studies on the altered metabolism of HMs have been mainly focused on AML and ALL. The available evidence supports the hypothesis that these leukemia subtypes demonstrate a dependence on glycolysis under aerobic conditions, thus providing a potential opportunity for the use of glycolysis inhibitors in acute leukemia. 

It has been published that leukemia cells, as tumor cells, shift their metabolism from oxidative phosphorylation toward the less efficient glycolysis. An evidence of this phenomenon comes from the observation that several genes involved in glucose metabolism were reported to be differentially expressed in pediatric leukemia. Expression profiling showed that the expression of HIF-1*α*, GLUT1, GLUT3, carbonic anhydrase 4 (CA4), and GAPDH was significantly higher in leukemic cells than in normal peripheral blood [109]. In addition leukemic cells with an increase of glycolytic rate appear to display glucocorticoid resistance, and the inhibition of glycolysis, rendered otherwise resistant leukemia cells susceptible to glucocorticoid treatment. The first generation glycolysis inhibitor, 2-DG, could reverse the glucocorticoid resistance in leukemia cells [110]. Treatment with 2-DG enhanced the chemotherapeutic effects of glucocorticoids (i.e., dexamethasone, prednisone and derivate) in particular against leukemic cells with mitochondrial defects. The phenomenon has been shown not only in cell lines but was also observed from primary leukemic cells of pediatric leukemia patients. However, clinical feasibility is limited with this compound [111] due to the high (mM) concentrations of the drug required for efficacy. In addition, recent studies indicated that 2-DG not only inhibits glycolysis and consumption of glucose to produce ATP or fatty acids but can also be metabolized though the pentose phosphate pathway (PPP). Moreover, 2-DG alters protein glycosylation, in particular by inhibiting N-glycosylation. Through the inhibition of N-glycosylation, 2-DG has been shown to kill cells in normoxia and to produce subsequent cellular stress. For this reason, other inhibitors of the enzyme HKII were tested in leukemia. 3-BrPA seems to have a greater potency but still requires a high dosage and has limited solubility and biodistribution. A report in human lymphoma and the AML cell line HL60 documented the induction of cell death upon treatment with 3-BrPA [112–114].

A recent study described a possible combinatorial treatment using an inhibitor of glycolysis (3-BrPA) and an inhibitor of electron transfer in the mitochondrial complex-III (antimycin A). Antimycin A is also a potent inhibitor of cytochrome-c-reductase. As a single agent, antimycin A was shown to induce apoptosis and to increase glycolytic rates [115, 116]. When 3-BrPA and antimycin A were combined, a dramatic decrease in the ATP levels of cancer cells was observed [117]. This study demonstrated that acute leukemia appears to be dependent on glycolysis for survival and that blockade of oxidative respiration can also have an effect on cell viability. However, the combined inhibition of glycolysis and oxidative phosphorylation led to more significant cell death. It is at present unclear whether this combinatorial treatment will also lead to toxicity in nonmalignant cells.

An alternative approach to potentiate the cytotoxic effects of glycolysis inhibitors in leukemia is the targeting of the mammalian target of rapamycin (mTOR) pathway, which is critical for cellular responses to metabolic stress [118]. The mTOR pathway plays an important role in nutrient uptake, regulation of energy metabolism, and cancer cells survival [118–120]. The targeted inhibition of both glycolysis and mTOR can cooperate to induce severe metabolic deregulation, cell death, and impaired ATP generation in cancer cells that are more dependent on glycolysis for energy production. *In vitro* and *in vivo* studies in leukemia and lymphoma cells [113, 120] demonstrated that a combination of rapamycin and 3-BrOP (3-bromo-2-oxopropionate-1-propyl ester), a cell permeable ester of 3-BrPA, effectively depleted cellular ATP [121]. Moreover, the combinatorial effect caused dephosphorylation of mTOR downstream target molecules (i.e., p70S6K) leading to alterations not only in cellular metabolism but also in survival signals. Furthermore, the simultaneous inhibition of glycolysis and the mTOR pathway is a potentially less toxic approach to target cancer cells. When ATP is depleted by glycolysis inhibition, blocking the mTOR patway further limits nutrient uptake, cell proliferation, and cell survival [121].

The sensitivity of leukemia cells to combinations of glycolytic inhibitors, such as 2-DG and 3-BrPA, and inhibitors of oxidative phosphorylation (antimycin A) or the mTOR pathway (rapamycin analogues) suggests a potential role for combinatorial therapeutic approaches in HMs. These combinations may lead to alterations not only in cellular metabolism but also in survival signal [122, 123].

Recent findings have suggested that the Warburg effect is also present in multiple myeloma (MM) [96]. For this reason, several research groups have tried to inhibit glycolysis as a novel therapeutic approach in these HMs. A recent study from Fujiwara et al. [124] described a new inhibitor of glycolysis, DCA, which promotes pyruvate oxidation and induced MM growth inhibition. The inhibition of aerobic glycolysis by DCA occurred via the downregulation of PDK1, a kinase that phosphorylates and inhibits PDH within the mitochondria. The inhibition of PDK1 and the restoration of the activity of PDH induced by DCA led to an increased supply of acetyl-CoA to the Krebs cycle and NADH electron donation to the electron transport chain [98]. An increase in electron transport chain activity causes a generation of mitochondrial reactive oxygen species (ROS) which contribute to generating a loss of mitochondrial membrane potential and ultimately the suppression of cell proliferation. 

The standard therapy for patients with MM is based on bortezomib, which induces a strong apoptotic response in myeloma cells [96]. However, plasma cells can become resistant to apoptosis, a phenomenon which is linked to aerobic glycolysis and predicts a poor clinical outcome in patients. Fujiwara et al. described that a combinatorial treatment of MM with bortezomib and DCA significantly increased superoxide production and induced apoptosis. In addition the authors performed an *in vivo* study with myeloma-bearing mice. A significantly prolonged survival of myeloma-bearing mice was observed upon treatment with the combinatorial therapy (DCA + bortezomib) [96], when compared with control mice or mice treated only with a monotherapy. Thus, inhibition of glycolysis increased the sensitivity of MM cells to conventional chemotherapy *in vitro* and improved the survival of myeloma-bearing mice *in vivo*. 

## 22. Conclusion

Targeting glycolysis has emerged as a potential novel approach to develop targeted therapies in HMs. However, additional studies are needed to investigate the molecular mechanisms of the dependency of HMs on glycolysis and its importance in chemoresistance. Since the use of cell lines from leukemia, lymphoma, or MM has certain limitations, such as metabolic adaptation and changes in the rates of cell growth and proliferation, it will be important to also test combinatorial strategies involving glycolytic inhibitors in primary samples from patients with HMs. If successful, these studies will warrant further investigations *in vivo* in appropriate models of HMs. Such work is a prerequisite for the successful transition of targeted therapies using inhibitors of glycolysis to perform clinical testing in patients with HMs.

## Figures and Tables

**Table 1:** Summary of reported alterations in proteins involved in metabolism in different types of cancer.

Protein	Alteration	Tumor type	Reference
GLUT1	mRNA overexpression	Endometrial and breast cancer	[19, 125]
HKII	mRNA overexpression	Glioblastoma multiforme	[126]
PGI	mRNA overexpression	Colorectal cancer	[31]
PFK1	mRNA overexpression	Breast cancer	[34–36]
GAPDH	mRNA overexpression	Lung, renal, breast, colorectal, hepatocellular, and pancreatic cancers	[127–129]
PKM2	mRNA overexpression	Lung, renal, breast, colorectal, and gastrointestinal cancers	[129–133]
LDH-A	Gene amplification	Lung, pancreatic, and colorectal cancers	[92]

GLUT1: glucose transport 1; HK: hexokinase; PGI: phosphoglucose isomerase; PFK: phosphofructokinase; GAPDH: glyceraldehyde-3-phosphate dehydrogenase; PKM2: pyruvate kinase M2; LDH-A: lactate dehydrogenase A.

Information was retrieved from http://www.ncbi.nlm.nih.gov/pubmed.

**Table 2:** Summary of preclinical studies and ongoing clinical trials with glycolysis inhibitors.

Drug	Target	Group or cell lines	Phase	Reference
2-DG	HK	Lung, breast, head and neck, pancreatic, and gastric cancers	Completed in 2009	[134]
2-DG	HK	Patients with advanced cancer and hormone refractory prostate cancer	Completed in 2011	[135]
3-BrPA	HK	Childhood acute lymphoblastic leukemia cell lines	Preclinical	[110, 121]
FX11	LDH-A	Tumor growth	Preclinical	[92]

2-Deoxyglucose (2-DG), 3-bromopyruvate (3-BrPA), (FX11 [3-dihydroxy-6-methyl-7-(phenylmethyl)-4-propylnaphthalene-1-carboxilic acid]) (FX11), and dichloroacetate (DCA). Information was retrieved from http://www.ncbi.nlm.nih.gov/pubmed.
